# Supplementary figures and images for: Characterization of the transcriptional divergence between the subspecies of cultivated rice (Oryza sativa)
Source: BMC Genomics. 2020 Jun 8;21:394. doi: 10.1186/s12864-020-06786-6 (PMC7278148; doi:10.1186/s12864-020-06786-6)

A

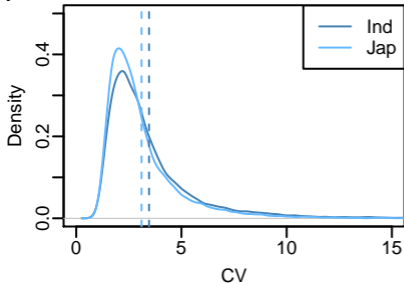

B

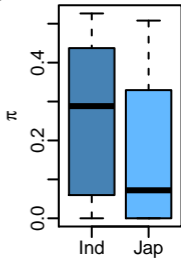

Supplement: Supplementary file 1 — Additional file 1 Genetic and expression diversity within Indica and Japonica accessions. [file 12864_2020_6786_MOESM1_ESM.pdf]

A

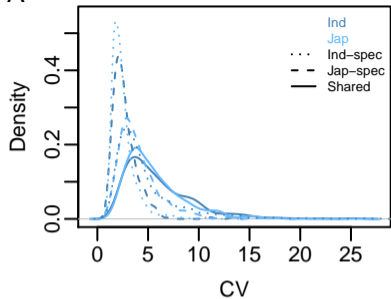

B

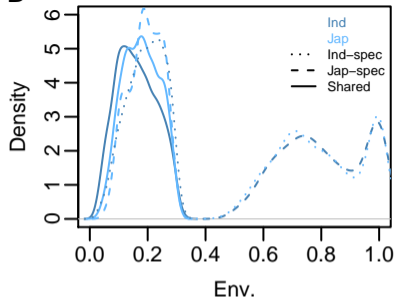

C

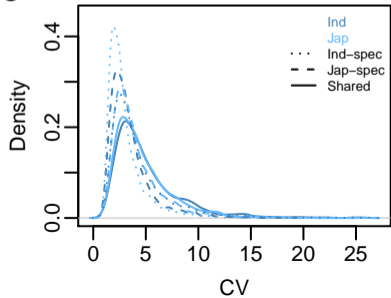

D

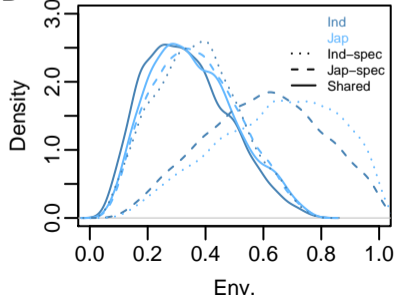

Supplement: Supplementary file 2 — Additional file 2 Assessing phenotypic variation and environmental effects for genes exhibiting genetic variability within each subspecies. [file 12864_2020_6786_MOESM2_ESM.pdf]
